# Copeptin levels and blood lipid profile in borderline patients with or without self-mutilation

**DOI:** 10.4102/sajpsychiatry.v22i1.976

**Published:** 2016-10-24

**Authors:** Sevda Korkmaz, Bilal Üstündağ, Ömer Özer, Gülay Taşç, Şüheda Kaya, Metin Ateşçelik, Murad Atmaca

**Affiliations:** 1 Department of Psychiatry, Fırat University Medicine Faculty, Turkey; 2 Department of Biochemistry, Fırat University Medicine Faculty, Turkey; 3 Department of Emergency, Fırat University Medicine Faculty, Turkey

## Abstract

**Purpose:**

Self-mutilation, known as self-harming behaviour of an individual without the intention of suicide, is commonly observed in individuals with borderline personality disorder. The objective of this study is to compare copeptin levels that are known to be related to aggressive behaviour and blood lipid profiles in borderline patients with and without self-mutilation.

**Methods:**

Twenty patients with self-mutilation [SM(+)] and 20 patients without self-mutilation [SM(−)] between the ages of 18 and 49, diagnosed with borderline personality disorder based on DSM-IV-TR(8) diagnostic criteria and attended to by Fırat University Psychiatry Polyclinic, participated in the study. Socio-demographic and clinical data form, Hamilton Depression Rating Scale, Hamilton Anxiety Rating Scale (HAMA) and Barrat Impulsivity Scale (BIS) were applied to all participants. Copeptin levels and plasma lipid levels were studied in the blood samples taken from the participants.

**Results:**

Mean copeptin level found in SM(+) group (37.54 ± 18.8 ng/mL) was statistically significantly higher than SM(−) group (18.53 ± 16.6 ng/mL) (*p* = 0.002). A negative correlation was found between mean copeptin and mean total cholesterol levels (*r* = −0.436; *p* = 0.005), and between copeptin and low-density lipoprotein cholesterol (LDL) levels (*r* = −0.403; *p* = 0.01) in both SM(+) and SM(−) patient groups. HAMA mean score for SM(+) group was found as 36.45 ± 13.2, and for SM(−) group, it was found as 35.7 ± 12.9. There was a statistically significant difference between the depression points achieved by the two groups (*p* = 0.046). BIS total points average for SM(+) group was determined as 71 ± 9.71, whereas it was determined as 66.8 ± 7.92 in SM(−) group. There was no statistically significant difference between the groups based on anxiety points. Barrat planning, Barrat motor and Barrat attention points for SM(+) group were higher than the SM(−) group. However, the difference was not statistically significant (*p* > 0.05).

**Conclusion:**

Findings of the study demonstrated that as cholesterol and LDL levels decreased, copeptin levels increased, and that could be related to the self-mutilation behaviour.

## Introduction

Borderline personality disorder is a psychiatric disorder that starts in early adolescence and is characterised by variations in inter-personal relationships, sense of self and mood swings.^1^ It is observed in 2% of the general population, and the incidence in females is three times that of in males.^2^ The disorder is characterised by psychiatric symptoms such as frequent changes in mood and also a continuous feeling of emptiness, unstable inter-personal relations, impulsive behaviour and agitation. Periodically, self-mutilation behaviour and suicide attempts are observed in patients because of intense anxiety and depression experienced as a result of stress. Self-mutilation is defined as a harmful behaviour of an individual directed at their own body without an intent of suicide.^3^ Self-mutilation is frequently seen in antisocial and histrionic personality disorders and especially in borderline personality disorder.^4^ Studies have suggested that serum cholesterol levels are significantly low in this patient group, and these low cholesterol levels could have a role in suicidal attempts.^5,6^ Also animal studies indicated that arginine vasopressin (AVP) molecule that has a role in endogen stress response was associated with aggressive behavior.^7^ In a study by Ramirez et al., it was reported that there was a relationship between AVP molecule and cholesterol levels; however, that relationship was observed only in males.^8^ Thus, we projected a possible relationship between AVP molecule, aggression and serum cholesterol levels. However, the authors could not find studies in the literature that investigated the AVP molecule or its precursor copeptin in borderline patients. Thus, it is not known whether there was a difference between copeptin and cholesterol levels amongst borderline patients with and without self-mutilation behaviour. We anticipated that the research of the correlation between self-mutilation that is known as the aggressive behaviour of an individual towards himself and copeptin and cholesterol molecules would contribute to the understanding of the aetiology of the said disease. Thus, we aimed to compare plasma copeptin and cholesterol levels in patients of borderline personality disorder with and without self-mutilation.

This study, therefore, aims to compare plasma copeptin and cholesterol levels in borderline patients with and without self-mutilation behaviour.

## Material and methodology

In accordance with the Helsinki Declaration, the study was commenced after obtaining the ethics committee approval. Twenty patients with self-mutilation and 20 patients without self-mutilation between the ages of 18 and 49, diagnosed with borderline personality disorder based on DSM-IV-TR^9^ diagnostic criteria and attended to by the Fırat University Psychiatry Polyclinic, participated in the study. Exclusion criteria were the use of a psychotropic agent (antidepressant, typical or atypical antipsychotic, mood stabilizer and anxiolytic) or a drug that could affect lipid metabolism during the last 2 weeks, history of cholesterol-lowering medicine, existence of an endocrinological disease, alcohol or substance use or addiction. Also, patients on steroid treatment and patients with renal failure were excluded from the study. A socio-demographic and clinical data form that was prepared by the authors in accordance with the information obtained through clinical experiences and the literature review, and in conformity with the aim of the study was applied to the patients. This was a semi-structured form including socio-demographic information, such as age, marital status, education, profession, gender, location of residence, economic standing and family status, and clinical information, such as duration of disease, number of hospitalisations and psychosocial stress factor on the onset of the disease. After a period of fasting for 12 h, blood samples were taken from all participating patients in two parts from the antecubital vein. For measurement of copeptin levels, 3 cubic centimetres blood samples were placed in aprotinin containing tubes; for blood lipid level measurement, 3 cc blood samples were placed in regular biochemistry tubes with gel. Blood samples were centrifuged for 5 minutes at 4000 rpm to separate into serum and plasma. Triglyceride (TG), total cholesterol (T kol), low-density lipoprotein cholesterol (LDL) and high-density lipoprotein cholesterol (HDL) levels were determined using Siemens Advia 1800 auto-analyser (Siemens Healthcare Diagnostics Inc., Tarrytown, NY, USA) and compatible kits (Advia Chemistry). After plasma samples were taken out of -20 ᵒC storage and thawed, they were studied with human copeptin ELISA kit (YH Biosearch Laboratory, lot number: 20150706 and Ref: yhb20150740650) within the kit operation procedure. Intra-assay coefficient of variability (CV) for the kit used in the study was < 10%, and its inter-assay CV value was < 12%, and the unit of measurement was ng/mL.

### Clinical scales used

DSM IV-TR Clinical Interview Form Structured for Personality Disorders (SCID II) was used in the study to support the borderline personality disorder diagnosis. SCID II is an individually applied structured form developed by Spitzer et al. to investigate personality disorders.^10^

Hamilton Depression Rating Scale (HAMD) was applied to all participating patients. HAMD score range was classified as 0–7 = normal, 8–13 = mild depression, 14–18 = moderate depression and 19–22 = severe depression.^11^ Also, Hamilton Anxiety Rating Scale (HAMA) was used to measure the severity of anxiety symptoms that could accompany the disease.^12^ Since impulsivity is commonly seen in borderline personality disorder patients, Barrat Impulsivity Scale (BIS) that consists of 30 items was applied to measure the impulsivity.^13^

### Statistical analysis

SPSS version 22 software package was used for statistical analyses. Normal distributions were tested with the Kolmogorov–Smirnov test with Lilliefors correction. Quantitative data were presented as mean ± standard deviation (s.d.), whereas nonparametric continuous values were presented as median (pp. 25–75). Student’s *t*-test was conducted for comparison data that displayed a normal distribution, and Mann–Whitney *U*-test was used for comparison data that did not display a normal distribution. Analysis of covariance (ANCOVA) was also used to adjust serum copeptin level for T kol, LDL and TG levels, and HAMA. *P* < 0.05 values were accepted as statistically significant.

## Findings

### Sample characteristics

In the study, socio-demographical characteristics of patients with self-mutilation [SM(+)] and patients without self-mutilation [SM(−)] were similar (Table 1). Although average age in SM(+) group was 23.7 ± 5.27, the average age in SM(−) group was 26.8 ± 6.1; however, the difference between the groups was not statistically significant. Mean HAMD score for SM(+) group was 12.6 ± 5.8, whereas the mean HAMD score was 16.7 ± 6.7 for the SM(−) group. Although 3 individuals in SM(+) group did not have depression, 9 individuals had mild, 6 individuals had medium and 2 individuals had high levels of depression. Although 1 in SM(−) group did not have depression, 6 individuals had mild, 3 individuals had medium and 10 individuals had high levels of depression. There was a statistically significant difference between the groups on depression scores (*p* = 0.046). HAMA mean score was found as 36.45 ± 13.2 in SM(+) group, and as 35.7 ± 12.9 in SM(−) group. There was no statistically significant difference between the groups based on anxiety scores.

**TABLE 1:** Comparison of socio-demographic characteristics of SM(+) and SM(−) groups.

Variable	SM(+) group *n* = 20)	SM(−) group *n* = 20
Mean age (years)(min–max)	23.7 ± 5.27(18–39)	26.8 ± 6.1(18–38)
**Marital status *n* (%)**		
Married	11 (55)	9 (45)
Bachelor	7 (35)	8 (40)
Divorced	2 (10)	3 (15)
**Education *n* (%)**		
Illiterate	-	2 (10)
Primary/secondary school	14 (70)	14 (70)
College	6 (30)	4 (20)
Residence centre *n* (%)	20 (100)	15 (75)
Alcohol users *n* (%)	1 (5)	1 (5)
Substance use *n* (%)	-	-
Smoking *n* (%)	10 (50)	11 (55)
Psychiatric disorders in family	12 (60)	16 (80)
Suicide history *n* (%)	20 (100)	-
Ekt performed *n* (%)	-	-
Psychiatric treatment history *n* (%)	11 (55)	20 (100)
Additional medical diseases *n* (%)	4 (20)	4 (20)
Employed *n* (%)	9 (45)	1 (5)
BMI (kg/m^2^), mean ± standard deviations	27 ± 4	28 ± 4
Weight (kg), mean ± standard deviations	72 ± 8	73 ± 9
Height (cm), mean ± standard deviations	163 ± 7	164 ± 8

BMI, body mass index; Ekt, electroconvulsive therapy.

BIS total points average was determined as 71 ± 9.71 in SM(+) group and was determined as 66.8 ± 7.92 in SM(−) group. There was a significant difference between the groups based on the BIS total points (*p* = 0.036). Barrat planning, Barrat motor and Barrat attention points were higher in SM(+) group when compared to SM(−) group. There was no statistically significant difference between the groups based on Barrat planning points (*p* = 0.054), Barrat motor points (*p* = 0.228) and Barrat attention points (*p* = 1.0).

Average total cholesterol level was 150.2 ± 26.9 mg/dl in SM(+) group. This value was determined as 181 ± 32.9 mg/dl in the SM(−) group. There was a significant difference between the groups based on the cholesterol levels (*p* = 0.003). LDL rates were significantly lower in the SM(+) group when compared to the SM(−) group (*p* = 0.007). In SM(−) group, both very low density lipoprotein and TG rates were statistically significantly higher than SM(+) group (Table 2).

**TABLE 2:** Comparison of clinical scale scores, copeptin levels and lipid parameters of SM(+) and SM(−) groups.

Variable	SM(+) group	SM(−) group	*p*
HAMD, mean ± s.d.	12.6 ± 5.8	16.7 ± 6.7	0.046*
HAMA, mean ± s.d.	36.45 ± 13.2	35.7 ± 12.9	0.857
BIS (planning), mean ± s.d.	26.2 ± 3.6	24 ± 3.4	0.054
BIS (motor), mean ± s.d.	26.6 ± 4.56	24.6 ± 5.7	0.228
BIS (attention), mean ± s.d.	18.2 ± 5.14	18.2 ± 3.4	1.0
BIS (total), mean ± s.d.	71 ± 9.72	66.8 ± 7.9	0.036*
Copeptin levels (ng/ml)			0.002†*
Mean ± s.d.	37.54 ± 18.8	18.53 ± 16.6	
Median (min–max)	46.8 (13.1–60.5)	10.7 (5.6 ± 54.6)	
Total cholesterol (mg/dl)			0.003*
Mean ± s.d.	150.2 ± 26.9	181 ± 32.9	
Median (min–max)	146 (105–203)	179 (133–259)	
LDL cholesterol (mg/dl)			0.007*
Mean ± s.d.	93.1 ± 27.1	117.4 ± 26.4
Median (min–max)	92.5 (40–145)	111 (76–169)
HDL cholesterol (mg/dl)			0.521*
Mean ± s.d.	47 ± 6.6	49.08 ± 12.8	
Median (min–max)	46.4 (35–61)	46.5 (34–82)
Triglyceride (mg/dl)			0.024^a^*
Mean ± s.d.	69.4 ± 32.8	130.9 ± 112.3	
Median (min–max)	60 (29–148)	110 (45–498)	
VLDL cholesterol (mg/dl)	14 ± 6.58	21.7 ± 14.6	0.038†*
Mean ± s.d.	12 (6–30)	20.5 (9–72)	
Median (min–max)		

HAMD, Hamilton Depression Scale; HAMA, Hamilton Anxiety Scale; BIS, Barrat Impulsiveness Scale; LDL, low-density lipoprotein; HDL, high-density lipoprotein; VLDL, very low-density lipoprotein; s.d., standard deviations.

† Mann–Witney *U*-test.

* *p* < 0.05.

### Copeptin and cholesterol and its relationship with self-mutilation

**FIGURE 1:**
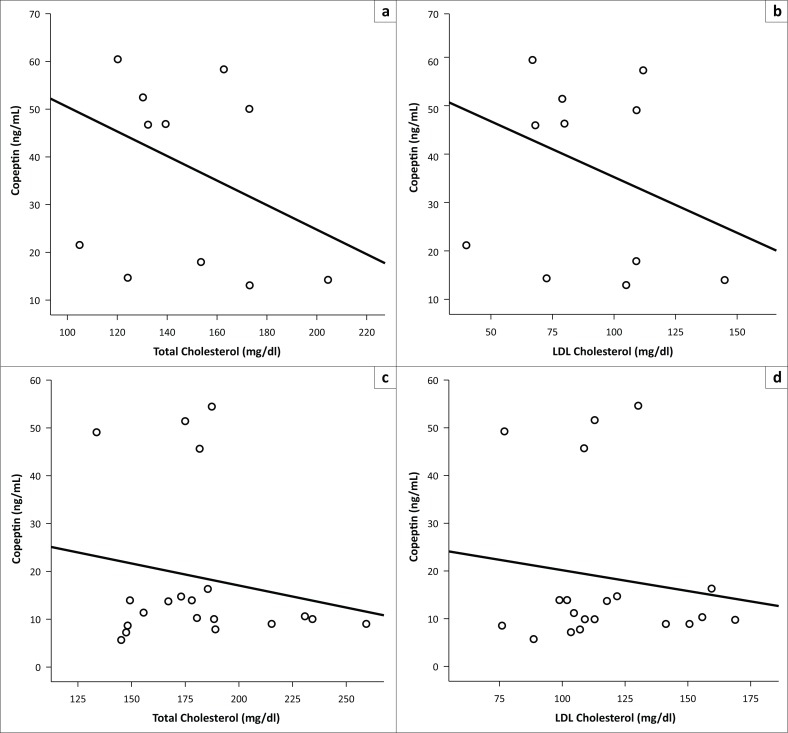
Correlation between serum copeptin and total cholesterol (a) and serum copeptin and LDL cholesterol levels (b) in borderline personality disorder [SM(+)]. Correlation between serum copeptin and total cholesterol (c) and serum copeptin and LDL cholesterol (d) in borderline personality disorder [SM(−)].

SM(+) group average copeptin levels (37.54 ± 18.8 ng/mL) were statistically significantly higher when compared to SM(−) group average copeptin levels (18.53 ± 16.6 ng/mL) (*p* = 0.002) (Mann–Whitney *U*-test was used). A negative correlation was indicated between the copeptin and cholesterol averages (*r* = −0.436; *p* = 0.005), and between copeptin and LDL levels (*r* = −0.403; *p* = 0.01) in both SM(+) and SM(−) patient groups (Figure 1).

There was no correlation between copeptin levels and Hamilton depression (*r* = −0.246; *p* = 0.126), Hamilton anxiety points (*r* = −0.031; *p* = 0.848) and BIS total points (*r* = 0.051; *p* = 0.755) for all study groups.

In addition, ANCOVA was used to adjust serum copeptin level for T kol, LDL and TG levels. The increases of copeptin level in the SM(+) group compared to the SM(−) group remained significant even after adjustments for total cholesterol, LDL cholesterol and triglyceride levels (35.16 ± 4.3 ng/mL vs. 20.91 ± 4.2 ng/mL; *p* = 0.032).

## Discussion

In the study, copeptin levels in patients with self-mutilation were found to be significantly higher than patients without self-mutilation. Copeptin is a precursor of AVP that plays a role in endogen stress response and reflects its plasma concentrations, and it is a stable and easily measurable peptide.^14^ AVP, also known as antidiuretic hormone, is a peptide released from the hypothalamus as a response to changes in plasma osmolality or arterial hypovolemia, and indicates endogen stress levels. Studies demonstrated that AVP amounts in cerebrospinal fluid changed in direct proportion with the aggressive behaviour, but changed inversely with the plasma serotonin levels.^15^ It was reported adverse early life events increased AVP gene expression and AVP release, correlating with an increase in suicide attempts and aggressive behaviour.^16,17^ Thus, it could be considered that copeptin, the precursor of AVP, was related to self-mutilation in individuals with borderline personality disorder. Findings of this study are consistent with the information that copeptin levels increase in individuals that have aggressive and self-mutilation behaviour. Thus, this study is significant for the fact that it was the first study in which copeptin molecule was studied in borderline patient population.

It was determined in the study that cholesterol and LDL levels were lower in SM(+) patient group when compared to SM(−) group. In fact, the relationship between the low serum cholesterol or the serum cholesterol reduced for treatment purposes, and impulsive and aggressive behaviour was studied extensively before.^18^ Also, the relationship between low serum cholesterol and impulsive and aggressive behaviour was scrutinised for a long period of time. There is even information that aggressive behaviour increased with the reduction in cholesterol levels in the field of anti-hyperlipidemic therapy.^18^ Studies have demonstrated that low serum cholesterol was related to suicidal behaviour in antisocial personality disorder^19^ and schizophrenia^20^ patients. It was found that the decrease in cholesterol levels reduced serotonin receptor sensitivity by altering the fluidity of neuron membranes. It was also reported that the decrease in cholesterol levels reduced 5-HT neurotransmission both in presynaptic and postsynaptic regions. It was suggested that there was a significant correlation between reduced central serotonin activity and aggressiveness and suicidal behaviour, especially in patients with personality disorders.^21^ It was reported that a decrease in cholesterol level disrupts the fluidity of neuron membranes and reduced both the serotonin sensitivity and 5-HT neurotransmission in postsynaptic regions. It was also suggested that there was a significant relationship between the reduced serotonin activity and aggressive and suicidal behaviour, especially in patients with personality disorders.^21^ In a study by New et al. that compared 14 patients with borderline personality disorder to 28 patients with other personality disorders, it was determined that serum cholesterol levels were significantly lower in borderline patients.^5^ The findings of this study are consistent with the results of the studies reported in the literature. A significant relationship between the copeptin levels and cholesterol and LDL rates was found in the study. Based on the findings, we could conclude that copeptin levels increased as cholesterol and LDL rates decreased, and this was related to the self-mutilation behaviour. Our findings showed that decreases in cholesterol and LDL levels were associated with an increase in copeptin levels, which might be related to self-mutilating behaviour.

The most significant limitation of this study is the lack of a healthy control group. However, it was considered significant to compare the patient groups based on the existence or non-existence of self-mutilation. In addition, the limited number of patients, female-only patient groups and the fact that blood samples were not taken during self-mutilation could be considered as the other limitations of this study. Furthermore, other limitations of the study are the small number of patients and the inability to take blood samples on the moment of self-mutilation. Because only female patients participated in the study, hormonal and menstrual factors could have affected the copeptin levels. Factors such as BMI, physical trauma and stress could also affect copeptin levels, and measurements could deviate slightly. And because self-mutilation is determined with the statements of the patient or the relatives of the patient, possibility of misinformation could be considered as another limitation of this study.

## Conclusion

Increased copeptin levels in borderline personality disorder, which is a disease where self-mutilation is observed quite often, could be related to agitated and aggressive behaviour. However, this result should be supported with future studies with larger sample sizes.
